# Quantification of the *Lamin A/C* Transcript Variants in Cancer Cell Lines by Targeted Absolute Quantitative Proteomics and Correlation with mRNA Expression

**DOI:** 10.3390/ijms20081902

**Published:** 2019-04-17

**Authors:** Wedad Saeed Al-Qahtani, Mai Abduljabbar, Entissar S. AlSuhaibani, Anas Abdel Rahman, Ahmad Aljada

**Affiliations:** 1 Department of Forensic Biology, Faculty of Forensic Sciences, Naif Arab University for Security Sciences, Riyadh 11452, Saudi Arabia; walqahtani@nauss.edu.sa; 2 Department of Zoology, Faculty of Science, King Saud University, Riyadh 11362, Saudi Arabia; ealsuhaibani@hotmail.com; 3 Newborn Screening & Biochemical Genetics lab, Department of Genetics, King Faisal Specialist Hospital and Research Center, Riyadh 11211, Saudi Arabia; mai_jabbar@kfshrc.edu.sa; 4 Department of Biochemistry and Molecular Medicine, College of Medicine, Alfaisal University, Riyadh 11533, Saudi Arabia; 5 Department of Chemistry, Memorial University of Newfoundland, St. John’s, NL A1B 3X7, Canada

**Keywords:** lamin A, lamin C, lamin AΔ10, lamin AΔ50 (Progerin), LC–MS/MS, MCF7, U937

## Abstract

*Lamin A/C* proteins have key roles in nuclear structural integrity and chromosomal stability. *Lamin A/C* cumulative protein expression of all variants is reported by semi-quantitative Western blotting. To date, there have not been specific antibodies for the individual *Lamin A/C* transcript variants. We developed a mass spectrometric approach for the quantification of *Lamin A/C* transcript variants. A signature peptide for each specific splice variant of *Lamin A/C* was selected. A LC–MS/MS assay based on the selected signature peptides and their labeled internal standards was established to measure the expression of *Lamin A/C* transcript variant concentrations. The method validation was carried out according to Food and Drug Administration (FDA) guidelines. The expression levels of the *Lamin A/C* transcript variants were measured in samples derived from MCF7 and U937 cell lines. RT-qPCR assay was also used to quantitate and compare the mRNA expression of splice variants of *Lamin A/C*. The established and validated method showed a great linearity, sensitivity, and precision. The different expressed *Lamin A/C* variants in different cell lines were measured and their levels were in concordance with qRT-PCR results. The developed method is reproducible, reliable, and sensitive for measuring different *Lamin A/C* transcript variants in different cell lines.

## 1. Introduction

The nuclear envelope which encloses the nucleus is comprised of two layers of membrane, separated by the intermembranous space. The nuclear envelope is underlined by the nuclear lamina, consisting of polypeptides called lamins, which interlink membrane proteins and heterochromatin [1]. Lamins are type V intermediate filament proteins classified as A and B types according to their sequence and structural configurations. The A-type is expressed in most differentiated somatic cells and the B-type in nearly all cells. The A-type lamins interact with B-type lamins in a largely uncharacterized assembly process to form the nuclear lamina. The A-type lamins, represented by lamins A and C, are products of the *lamin A/C* gene generated by alternative splicing [2]. Recent unraveling of the complete human *lamin A/C* gene has provided a better insight into the mechanism by which lamins A and C are generated from the same gene [3]. The *lamin A/C* gene has 12 exons, alternative splicing of which creates different lamin isoforms. There are five known transcript variants of the *Lamin A/C* gene: Lamin A, lamin C, progerin (lamin AΔ50), lamin AΔ10, and lamin C2 (expressed in the testes). Lamins play a crucial role in the physical pairing of the nucleus with the cytoskeleton [4]. Being an integral component of the nuclear matrix, lamins facilitate chromatin organization, mediate attachment of various transmembrane proteins, and are involved in transcriptional activities, DNA replication, and repair processes [5,6]. Mutations in the *lamin A/C* gene have been linked to various disorders, known as laminopathies. *Lamin A/C* mutations have been linked to numerous heritable disorders, including cardiomyopathy, Emery-Dreifuss muscular dystrophy, neuropathy, lipodystrophy, cerebellar disorders, and premature aging syndrome [2,7,8].

Absolute quantification (AQUA) of specific proteins using MS-based methods has been developed to determine protein concentrations in many other studies using triple quadrupole mass spectrometry with multiple reaction monitoring (MRM), which is the most common method for quantitation of analyte by LCMSMS [9,10]. The signature peptide for the targeted protein is usually selected based on several criteria such as the targeted protease, peptide length, inter- and intra-species uniqueness, potential sites for post-translational modification (PTM), and history of being detected using mass spectrometry. Several bioinformatics tools could be utilized for this selection such as Skyline, BLAST, and Peptide Atlas. The targeted proteins from biological samples are cleaved using specific enzymes (e.g., trypsin) for the release of signature peptide fragments with unique primary sequences (signature peptides). These signature peptides are utilized as surrogates for corresponding parent proteins so that the small molecular peptides can be quantified to estimate the protein concentrations. This approach has been used in different research areas such as occupational asthma [11,12], or in the diagnosis such as inborn errors of metabolism, pancreatic cancer and oral cancer [2,13,14,15].

Most of the published studies have detected the proteins of *Lamin A/C* gene transcript variants as one single protein, without attempting to describe the translated transcript variants as separate proteins with separate biological functions. The basic objective of this study was to establish a multiplex sensitive method to quantify the four transcript variants of the *Lamin A/C* gene to allow the dissection of the different biological roles of these four transcript variants. Moreover, an AQUA tandem mass spectrometry method is developed using multiple reaction monitoring (MRM) and validated to assay the different *Lamin A/C* variant proteins using unique signature peptides (except for lamin A, which was calculated). This method represents an alternative, sensitive, and specific way to quantify proteins of *Lamin A/C* transcript variants compared to traditional Western blotting or immunohistochemistry (IHC) methods.

## 2. Results

### 2.1. Method Development for Lamin A/C Gene Transcript Variants and Mass Spectrometry Optimization

**Figure 1:**
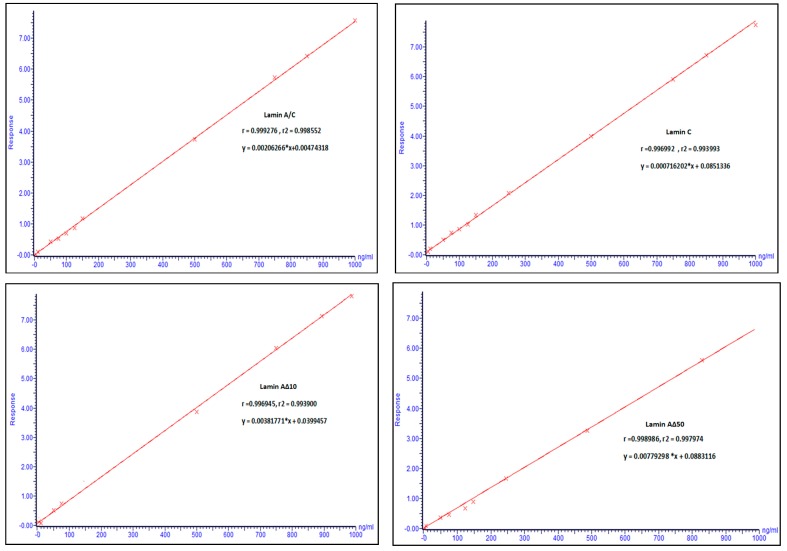
Calibration curves of optimized unlabeled peptides (standards) were spiked with internal standard (IS) and quantified by the LC–MS/MS method.

**Figure 2:**
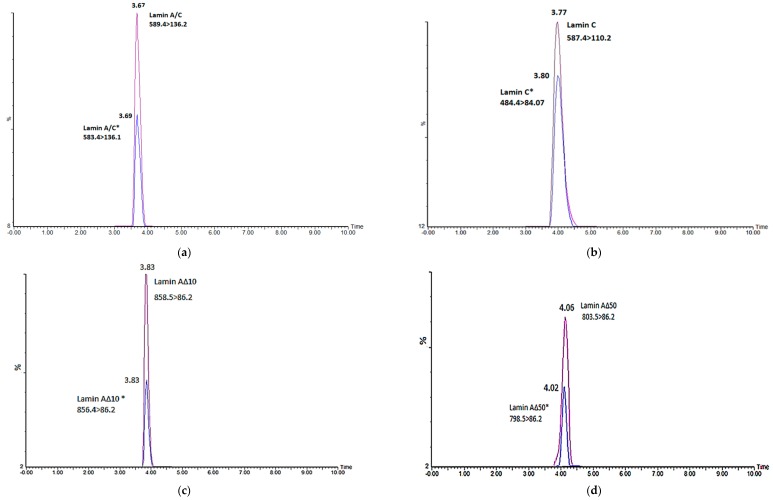
Chromatograms of optimized unlabeled peptides that were spiked with IS (*) at a concentration of 250 µL/mL. (**a**) Lamin A/C, (**b**) lamin C, (**c**) lamin ΔA10, (**d**) and lamin ΔA50 (Progerin).

In the present study, two sets of peptides (signature peptides (standards) and isotope-labeled peptides (internal standards)) were selected for each *lamin A/C* gene transcript variant. As we targeted transcript variants of *lamin A/C*, the signature peptides were selected to be generated after tryptic digestion with a unique region to each of the *Lamin A/C* transcript variant. Luckily, these signature peptides do not have any potential site for PTM such as cystine and have good size and history to be detected using mass spectrometry [16,17]. A mixture solution of the four signature peptides and their labeled analogies was used to optimize the LC–MS/MS. Initially, the ion source and triple quadrupole analyzer were optimized to generate the most abundant precursor and product ion for each peptide at the optimum cone voltage and collision energy to be able to construct the MRM transitions. The developed MS method was used to build the liquid chromatography method, where the peptides were separated in a reversed phase chromatography using a gradient elution within 10 min of running time (Table 1). A standard calibration curve for each peptide was constructed as shown in Figure 1 based on good symmetrical peaks (Figure 2). The optimum LC–MS/MS method parameters are summarized in Table 2. The mass parameters were fine-tuned for maximum sensitivity, and parent ion transitions were selected to afford the best response for the spectrum analysis. The method was validated using these optimized conditions.

### 2.2. Data Validation 

#### 2.2.1. Intra- and Inter-Day Accuracy and Precision

Under optimized LC–MS/MS conditions, samples extracted from cells showed no significant interfering peaks at the retention times for each signature peptide (Table 2). For linearity evaluation, three different calibration curves were prepared for each signature peptide on three consecutive days. Blank, blank with internal standard (IS) and 10–11 calibration curve points were analyzed using the developed method. The calibration curves were drawn by plotting the peak area ratio of analyte to IS versus the nominal concentration of each analyte. Six replicates of each peptide at nominal concentrations of 150, 300, and 600 ng/mL were used in three consecutive days of validation for the single assay (Table 3). The accuracy and precision of the method for determining lamin concentrations were within acceptable ranges according to United States Food and Drug Administration (US-FDA) and European Medicines Agency (EMA) guidelines, indicating the suitability and reproducibility of the analytical method at the specified concentration range. The intra-assay precision percent (CV%) ranged between 0.76% (observed for lamin AΔ50 on day 2) to 14.38% (observed for lamin C on day 1). Inter-assay precision percent (CV%) was in the range of 1.51% (observed for lamin AΔ50 with 600 ng/mL) to 11.66% (observed for lamin C with 150 ng/mL). All CV values for intra- and inter-day assays were ≤15%. All CV and accuracy (RE) values were within acceptable ranges according to US-FDA guidelines.

#### 2.2.2. Linearity, Sensitivity, and Stability

The sensitivity of the method was evaluated as described in the methodology section. Sensitivity was estimated for all four signature peptides, which ranged from 48.47 to 60.20 nM (maximum CV% of 14.62% recorded for lamin AΔ10). Moreover, the method linearity was evaluated for each signature peptide, where the coefficient of the determination (R^2^) ranges from 0.9726 to 0.9969 (Table 4).

The developed method for *lamin A/C* gene transcript variants was evaluated for short-term (at room temperature or 4 °C for 1 week), intermediate-term (at −20 °C for 2 weeks), and long-term (at −20 and −80 °C for one month) stability. Stability was determined in three replicates at three levels of Quality Control (QC) (150, 300, and 600 ng/mL). Stability analysis indicated that lamins were stable for up to 1 month in extracted protein at −20 °C (Table 5). All CV and RE values for each stability condition tested were ≤5%.

Intra-assay precision percent (CV%) ranged between 0.76% (observed for lamin AΔ50 on day 2) to 8.34% (observed for lamin C on day 2), and accuracy percent (RE%) ranged from 12.21% (observed for lamin A/C on day 2) to 14.51% (observed for lamin AΔ50 on day 1).

Inter-assay precision percent (CV%) was in the range of 1.38% (observed for lamin A/C and lamin AΔ50) to 6.13% (observed for lamin C), whereas accuracy percent (RE%) ranged from 4.61% (observed for lamin AΔ10) to 8.56% (observed for lamin A/C). All CV and RE values for intra- and inter-day assays were ≤15%. These results indicate that all CV and RE values are within acceptable ranges according to US-FDA guidelines.

### 2.3. Validation of Mass Spectrometric Method by Recombinant Lamin A/C Gene Transcript Variants

**Figure 3:**
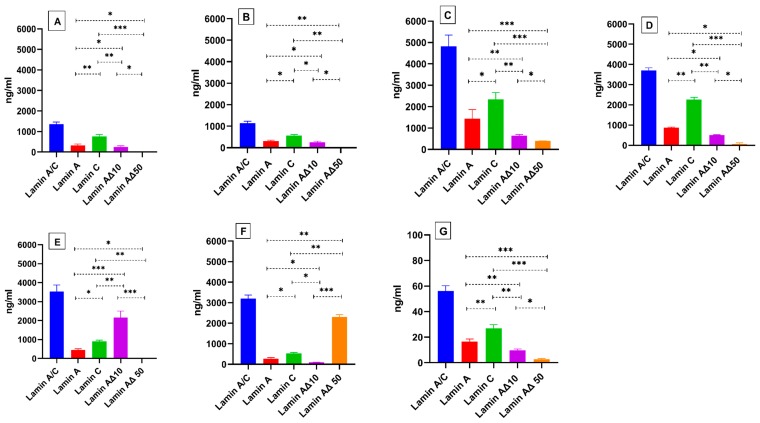
Protein quantitation of total lamin A/C and calculated lamin A, lamin C, lamin AΔ10, and lamin AΔ50. Differential protein expression levels of *lamin A/C* transcript variants were measured in (**A**) the non-transfected MCF7 cell line, (**B**) the PCMV6-XL4 transfected MCF7 (Mock transfection), and following transfection of MCF7 cells with cDNA coding for (**C**) lamin A, (**D**) lamin C, (**E**) lamin AΔ10, and (**F**) lamin AΔ50. Total lamin A/C and calculated lamin A, lamin C, lamin AΔ10, and lamin AΔ50 were also measured in (**G**) the U937 cell line. Values are expressed as mean ± standard error (*n* = 9). Statistical comparisons of significant differences between groups with adjoining lines: * *p* < 0.01, ** *p* < 0.001, *** *p* < 0.0001.

**Figure 4:**
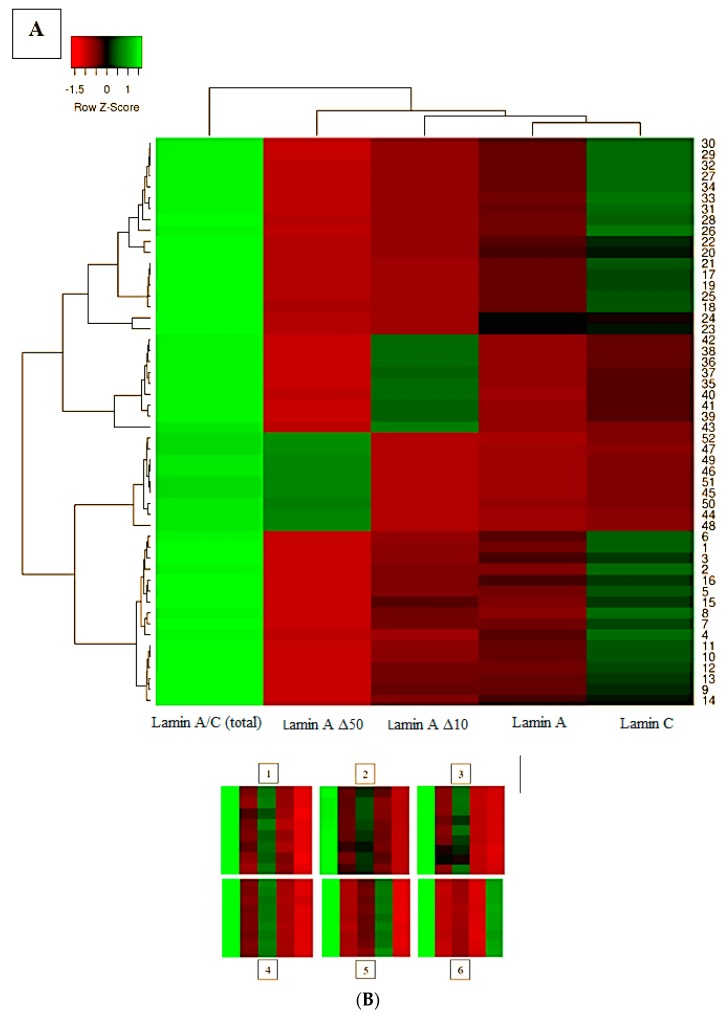
MCF7 cell lines. (**A**) Heat maps showing a two-way comparison of differential expression of lamins in transfected MCF7 cells (where MCF7 exhibits normal lamin expression). PCMV6-XL4 (MCF7 transfected with only PCMV6-XL4 vector with normal lamin expression), lamin A/C (total), lamin C, lamin AΔ10, and lamin AΔ50 detected using LC–MS/MS, *n* = mean of 9 replicates. (**B**) Selected portions of lamin expression levels in MCF7 cells (PCMV6-XL4). Lamin A/C (total), lamin A, lamin C, lamin A Δ10, and lamin A Δ50 are indicated by 1, 2, 3, 4, 5, and 6 respectively, which exhibit five concentrations of lamins from light green (high expression level) to red.

The protein expression levels of the various lamin splice variants (lamin A/C, lamin A, lamin C, lamin Δ10, and lamin Δ50) were significantly higher (*p* ≤ 0.001) in the transfected cells. Expression of lamin splice variants in U937 monocytes was significantly lower than in MCF7 (Figure 3). We detected no correlation between protein expression levels of *lamin A/C* transcriptvariants and tested cells (Figure 4).

### 2.4. Validation of Lamin A/C Gene Expression by RT-qPCR

**Figure 5:**
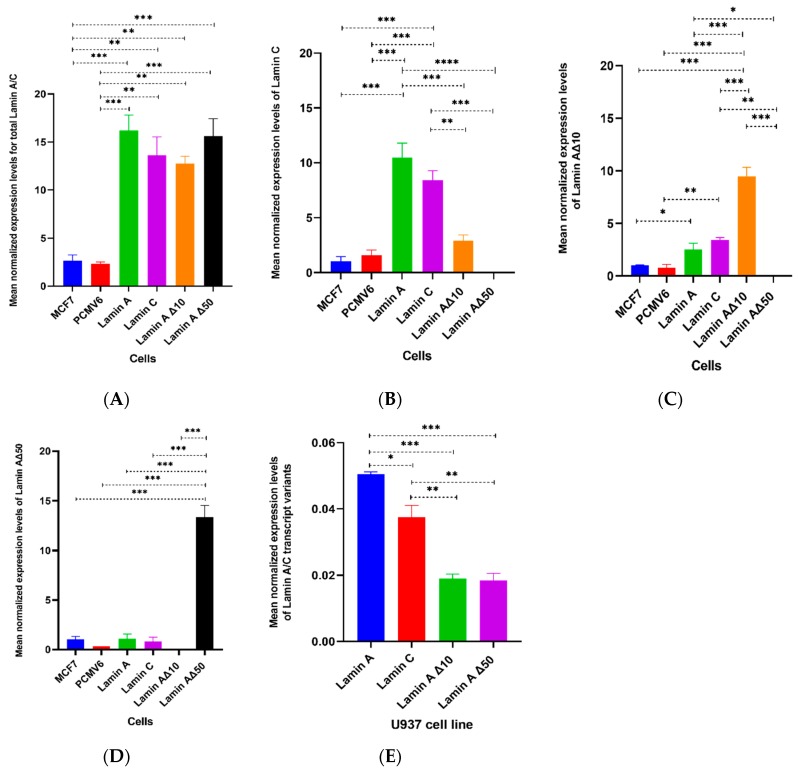
Differential mRNA expression levels of *lamin A/C* transcript variants: (**A**) Total *lamin A/C*, (**B**) *lamin C*, (**C**) *lamin AΔ10*, and (**D**) *lamin AΔ50*. mRNA expression levels were measured in native MCF7 and following transfection with PCMV6-XL4 plasmid (Mock transfection) or cDNA coding for lamin A, lamin C, lamin AΔ10, or lamin AΔ50 using RT-qPCR. Expression levels are expressed relative to non-transfected MCF7 expression levels. Differential mRNA expression levels of *lamin A*, *lamin C*, *lamin AΔ10*, *lamin AΔ50* were also measured in (**E**) the U937 cell line. Values are expressed as mean ± standard error (*n* = 9). Statistical comparisons of significant differences between groups with adjoining lines: * *p* < 0.01, ** *p* < 0.001, *** *p* < 0.0001, **** *p* < 0.00001.

An RT-qPCR protocol was used to validate *lamin A/C*, *lamin C*, *lamin AΔ10*, and *progerin* variants and to measure their relative abundances in MCF7 and U937 cell lines. The expression levels of *lamin A* and *lamin C* were higher than those of *progerin* and *lamin AΔ10*, which is consistent with our previous findings (Figure 5).

## 3. Discussion

The number of laminopathies is large, and their variability is equally wide. The multiplex mass spectrometric assay established in this study represents the first bioanalytical method to evaluate and quantify the *lamin A/C* gene transcript variants individually. This analytical method will likely be useful in efforts to dissect the biological role of the four *lamin A/C* transcript variants and their roles in laminopathies. Unraveling the specific biological role of each *lamin A/C* transcript variant could provide a better insight into the pathophysiology of laminopathies. Most of the published studies have detected *lamin A/C* gene transcript variants as one protein, without considering these transcript variants as separate proteins with separate biological functions. Moreover, the LC–MS/MS method represents a more sensitive and specific approach to quantifying proteins than traditional semi-quantitative troublesome Western blotting or relative quantitation of RT-qPCR. Currently, there is no specific antibody for lamin AΔ10 and attempts to raise a specific antibody for it have failed [18]. The antibody for lamin Δ50 lacks sensitivity, and the only methods to quantitate lamin A and lamin C are semi-quantitative Western blotting or IHC. The stability of RNA would also represent an obstacle for the routine use of the *lamin C*/*lamin A* mRNA ratio as a diagnostic test, as has been proposed by [19]. Furthermore, our novel method can be used to investigate the various transduction pathways involved in laminopathies associated with these four transcript variants.

The results obtained from LC–MS/MS are comparable with the results from RT-qPCR, which is a relative quantitative method of mRNA expression for *lamin A/C* transcript variants. However, our developed method is a quantitative method that allows us to quantitate each transcript variant of *lamin A/C*, especially *lamin A*, which is not calculated by RT-qPCR. Additionally, lamin A and lamin C have high abundance, whereas lamin AΔ10 and progerin have low abundance in the cells (MCF7 and U937) used in this study. At the same time, the four transcript variants of *lamin A/C* in U937, which is a monocytic cell line, have lower values compared to in MCF7 cells. Low levels of lamin A/C expression have been reported in monocytes [8,11,20,21]. Moreover, inactivation of the *lamin A/C* gene by CpG island promoter hypermethylation has been associated with leukemic monocytes. Our findings revealed that the developed method, using the selected signature peptides on LC–MS/MS, was able to detect expression of the four transcript variants of *lamin A/C* in leukemic monocytes of the U937 cell line. Interestingly, RT-qPCR could not detect *lamin AΔ10* or *lamin AΔ50* in mononuclear cells of normal or leukemic patients (unpublished data). Thus, this method could be a useful diagnostic tool for leukemia through quantitation of *lamin A/C* transcript variants ratios in monocytes. Moreover, the results showed an inverse correlation between lamin AΔ10 and progerin expression, where progerin induction inhibited lamin AΔ10.

Previous reports have indicated that *lamin A/C* transcript variant proteins are highly expressed in well-differentiated cells and tissues but are poorly expressed in stem cells [17]. Our findings revealed that *lamin A/C* transcript variant proteins are expressed instead of being completely absent in breast cancer or monocytic cell lines. The differences between our current findings and previous reports might be attributed to the methods used. This demonstrates the significance of the established LC–MS/MS sensitivity. The quantification of progerin expression by our sensitive and specific method might also allow for exploration of low levels of progerin in aging and other diseases [21,22,23]. Interestingly, lamin C and progerin have been shown to have distinct and opposite functions regarding energy expenditure and lifespan [24,25,26,27]. Thus, measurement of these transcript variants would likely contribute to understanding their role in energy expenditure.

Lamin A splice variants have been identified as possible biomarkers for cellular aging. Lamin A splice variants have also been implicated in premature aging disorders, such as Hutchinson-Gilford progeria syndrome (HGPS) in children and Werner syndrome in adults [16,28,29], as well as in muscular dystrophy, lipodystrophies, and cardiomyopathies [2,7,30,31]. The method presented in this paper was developed to facilitate examination of the biological and diagnostic potential of *Lamin A/C* transcript variant proteins. This LC–MS/MS bioanalytical method, which was validated according to international guidelines, showed extremely good selectivity with minimal matrix effects, and good linearity, accuracy, precision, recovery, and stability. The developed separation method produced acceptable values of recovery. The developed chromatogram has a well-resolved peak of *Lamin A/C* transcript variant proteins without interference and could be easily applied in studies of human laminopathies. The sensitivities for each *Lamin A/C* transcript variant protein varied over a wide range, as they depend on molecular proton affinity under common mass spectrometric parameters such as ion source, gas pressure and temperature, collision energy, and post-source chemical stability. Under optimum chromatographic conditions, peaks of targeted *Lamin A/C* transcript variant proteins were adequately separated at retention times of between 9 and 10 min.

## 4. Materials and Methods

### 4.1. Chemicals and Materials

MCF7 and U937 cell lines were purchased from the American Type Culture Collection (ATCC, Manassas, VA, USA). Ammonium sulfate, acetonitrile, hydrochloric acid, and methanol were supplied by ACP (Montreal, QC, Canada). Trypsin sequencing-grade enzyme was purchased from Promega (WI, USA). Tris (hydroxymethyl)aminomethane (Tris), dithiotheritol (DTT), ethylenediaminetetraacetic acid (EDTA), formic acid (FA), ammonium bicarbonate, o-methylisourea hemisulfate, ammonium hydroxide, horseradish peroxidase (HRP), chemiluminescent substrate, sodium dodecylsulphate (SDS), ammonium formate, and α-cyano-4-hydroxycinamic acid (HCCA) matrix were purchased from Sigma-Aldrich (St. Louis, MO, USA). Lipofectamin 3000 was obtained from Invitrogen, USA. Pierce BCA Protein Assay Kit (cat. no. 23225) was purchased from Pierce Chemical Co. (Rockford, IL, USA). RapiGest SF surfactant was purchased from Waters Corporation (Milford, MA, USA). The signature peptides, *lamin A/C* (purity > 98.91%; molar mass = 11,657.0 Da) and its deuterated isotopic homolog using d3-l-alanine (purity > 96.09%; molar mass 1177.2 Da), lamin C (purity > 98.67%; molar mass = 2899.8 Da) and its deuterated isotopic homolog using d8-l-valine (purity > 96.52%; molar mass 2932.3 Da), lamin AΔ10 (purity > 95.56%; molar mass = 2566.0 Da) and its deuterated isotopic homolog using d3-l-alanine (purity > 95.18%; molar mass 2572.1 Da), and lamin AΔ50 (purity > 95.77%; molar mass = 1595.4 Da) and its deuterated isotopic homolog using d3-l-alanine (purity > 99.57%; molar mass 1604.7 Da) were purchased from GeneMed Synthesis (San Francisco, CA, USA). All chemicals used in this study were of analytical grade.

### 4.2. Establishing a Multiplex Assay for the Simultaneous Quantification of the Four Lamin A/C Gene Transcript Variants

#### 4.2.1. Selection of Signature Peptides Specific for *Lamin A/C* Transcript Variants

**Figure 6:**
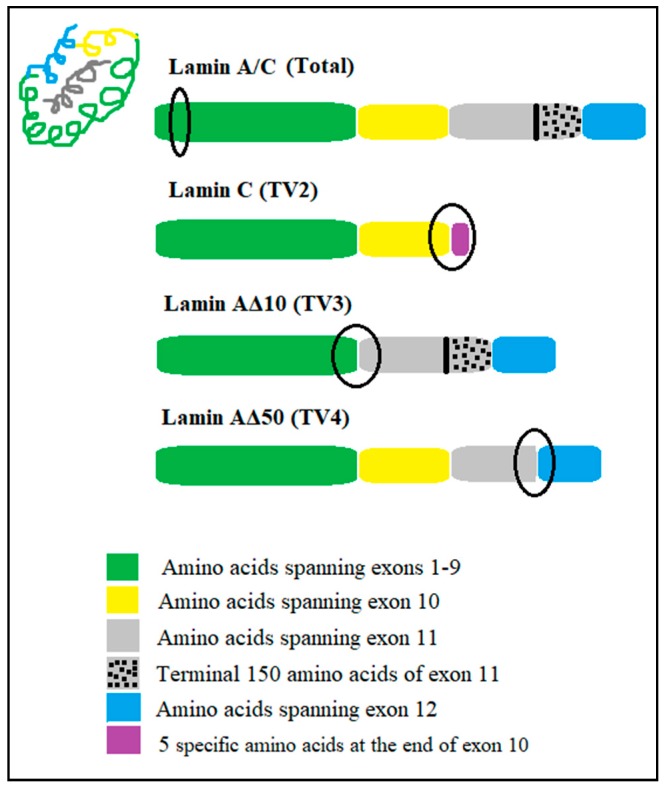
Locations of signature peptides on *lamin A/C* exons, marked by circles.

Using ExPASY: SIB Bioinformatics Resource Portal (http://www.expasy.org), signature tryptic peptides for *lamin A/C* gene transcript variants (lamin A/C, lamin C, lamin AΔ10, and lamin AΔ50) were selected. The signature peptide amino acid sequences for *lamin A/C* gene transcript variants, together with their location, are described in Figure 6 and Table 5. The goal was to select signature peptides that are between 5–20 amino acids in length, are free of potential sites for post-translational modification, can be cleaved efficiently by trypsin, and would be easily analyzed by mass spectrometry [32]. As selecting a signature peptide for lamin A is not possible, the total *lamin A/C* gene signature peptide targeting exon 1 was used to quantify all transcript variants. Then, subtraction of lamin C, lamin AΔ10, and lamin AΔ50 from the total *lamin A/C* proteins value gives the lamin A protein quantity. For lamin C, a signature peptide spanning the end of exon 10 having five amino acids that are specific for the end of lamin C was used. The lamin AΔ10 signature peptide spanning between exon 9 and exon 11 was also utilized, as lamin AΔ10 is missing exon 10. A signature peptide for progerin (lamin AΔ50) spanning the junction region of the exonic cryptic site of exon 11, which lacks the terminal 150 amino acids, and exon 12 was utilized (Table 6).

Small molecular quantification using MS methods involves an isotope dilution strategy that has been recognized as a reference method for internal standardization for absolute protein quantification. The calibration and routine samples are spiked with defined amounts of a stable isotopic analog(s) of unique peptides to establish the calibrating curves and give the absolute amount. All unlabeled and isotope-labeled peptides were custom synthesized by Genemed Synthesis, Inc (San Antonio, TX, USA).

#### 4.2.2. Establishing a Mass Spectrometric Method for *Lamin A/C* Gene Transcript Variants

The LC–MS/MS Xevo TQD-Triple-Quadrupole-Mass- Spectrometry system used in this project was from Waters (UPLC) and MSMS (Xevo TQD).

##### LC/MS Conditions

The peptide mixtures were eluted by reversed-phase chromatography on a Waters Acquity UPLC BEH C18, 2.1 × 50 mm, 1.7 µm column maintained at 25 °C. Solvent A was 0.1% Acetic acid and solvent B was a 1:1 mixture of methanol and acetonitrile. Standards and samples were injected in volumes of 10 µL. The flow rate throughout the method was set to 0.2 mL/min, and the individual peptides were separated by gradient elution. The gradient profile for solvent B was as follows: 10% for 1 min followed by a linear gradient to 90% over 5 min, which was then held at 90% for 0.2 min before returning to 10% in 0.3 min at 6.5 min post-injection. The column was equilibrated at 10% solvent B for 3.5 min before performing a second injection. Therefore, the total run time of the method was 10 min. Mass spectrometric detection of the peptides was performed by electrospray ionization (ESI) on a Xevo TQD MS/MS system operating in a positive ionization mode. The ionization parameters were as follows: Desolvation temperature, 500 °C; gas flow rate 1000 L/h; and source temperature 140 °C. A multiplexed MRM approach was used in the detection of the peptides. The precursor and product ions of the individual MRM transitions for each peptide and its isotope-labeled surrogate, the cone voltages, and collision energies are listed in Table 2. For each peptide, a multiplexed MRM approach that monitors the transition of the precursor ion to three signature product ions was used to ensure the specificity of the method for the target peptides. In optimizing the MRM transitions of the peptides, target analyte fragmentation was minimized while maximizing the fragmentation of the isobaric components. This approach allows the precursor ion of the analyte to pass the collision cell with minimum loss of signal intensity. The first minute into the chromatographic run was diverted into waste to avoid any possibility of ion suppression.

##### Preparation of Calibration Standards and Quality Control (QC) Samples

To prepare standards for calibration curves and quality control (QC) samples, a stock mixture of the unlabeled peptides was prepared such that each peptide was at a concentration of 1 mg/mL. Similarly, a stock mixture of the labeled peptides was prepared to a concentration of 0.25 mg/mL for each peptide. Calibration standards for the unlabeled peptides were prepared by serial dilution of the stock mixture to achieve the following target concentrations: 1, 5, 10, 25, 100, 150, 250, 500, 1000, 2500, and 5000 ng/mL. Each calibration standard was then spiked with 20 µL of the stock mixture of labeled peptides. Analytical QC samples were similarly prepared by spiking 20 µL of the stock mixture of labeled peptides to the serial concentrations of 150, 300, and 600 ng/mL of the unlabeled peptides.

##### Validation of the LC/MS Method

Validations of the analytical method for accuracy, precision, linearity, dynamic range, specificity, and sensitivity were performed according to the United States Food and Drug Administration (US-FDA) and International Conference on Harmonization (ICH) guidelines [33]. Calibration standards in neat solution for each signature peptide were prepared fresh over three consecutive days and were used in the construction of the calibration curve. The calibration curves for the signature peptides were generated by plotting the average peak area ratio (area of the unlabeled peptide/area of the labeled peptide) of the target peptide from three independent preparations versus the nominal concentration of the peptide. Method accuracy, linearity, range, specificity, and sensitivity of the peptides were estimated from the average of three calibration curves. Accuracy was calculated as the mean measured concentration/nominal concentration × 100%, and the variability in the concentration was determined by calculating the percentage relative standard deviation (%RSD). The lower limit of quantification (LLOQ) was determined at a signal intensity that was at least 10 times greater than the standard deviation of the blank, and its accuracy was set to a range of 80–120% and the day-to-day variation to less than or equal to 20%. The accuracy of all other standard levels in the calibration curve was set to a range of 85–115% and day-to-day variation that is less than 15%. The intra-day precision was evaluated using six replicates of each QC sample (150, 300, and 600 ng/mL), and was freshly prepared over the next two days for a total of 18 replicates to evaluate inter-day variation.

##### Stability

Stability of the samples was assessed using three replicates of QC samples at three concentrations (150, 300, and 600 ng/mL). The stability of labeled and unlabeled peptides was investigated under five different conditions. Two of the conditions were short-term periods, one at room temperature and other at 4 °C for 1 week. One of the conditions was the intermediate term for 2 weeks at −20 °C, and the remaining two conditions were long-term (freezing at −20 or −80 °C for 1 month).

### 4.3. Validation of the LC/MS Method by Recombinant Lamin A/C Gene Transcript Variants

The MCF7 cell line was transfected with pCMV6-XL4-expressing vector containing C-terminal GFP-tagged human full-length cDNA clones of *lamin A*, *lamin AΔ50*, *lamin AΔ10*, or *lamin C* (Origene Technologies, Inc., Rockville, MD, USA). Control cells were transfected with pCMV6-XL4. Transfection efficiency was evaluated by co-transfecting pCMV6-XL4-GFP and counting the number of cells transfected relative to the total number of cells. This method had 60–70% transfection efficiency in the breast cancer cell line used in this study. Cells were transfected using Lipofectamine 3000 (Invitrogen, Carlsbad, CA, USA). Stably transfected cells were selected with G418 for 14 days. Transfected MCF7 cells were characterized by fluorescence microscopy.

Total cellular protein was collected using 2 × 10^6^ mammalian cells. In brief, cells were suspended in 300 µL of 1× phosphate buffered saline (PBS) followed by washing three times with 2.0 mL of ice-cold 1× PBS. Pellets were centrifuged at 10,000 rpm for 2 min followed by addition of 500 µL of lysis buffer (10 mM Na3PO4; pH 7.0; 0.5% SDS) with gentle agitation to resuspend the cells. The cells were sonicated three times (60 s each time) on ice. The Pierce BCA protein assay was performed to check protein concentration (as per manufacturer’s instruction).

All prepared fractions of cellular protein were diluted to 500 µg/0.5 mL prior to the digestion process, and then 100 µL of each sample was subjected to freeze-drying. The dried samples were then dissolved in 100 µL of RapiGest SF solution that was prepared in accordance with the manufacturer instructions by dissolving 1 mg of RapiGest SF powder in 1000 µL of 50 mM Ammonium Bicarbonate at pH 7.8. To reduce the disulfide bonds in the protein fractions, dithiothreitol (DTT) was added to a final concentration of 5 mM, and samples were incubated at 50 °C for 30 min. Then, samples were cooled to room temperature, and iodoacetamide (IAA) was added to a final concentration of 15 mM. Samples were then incubated in the dark at room temperature for 30 min. Protein digestion was performed by adding trypsin to a total protein ratio of 1:50 (1 mg of trypsin was dissolved in 1 mL of 50 mM of Ammonium Bicarbonate; pH 7.8). Samples were digested by incubation at 37 °C overnight. At the end of the digestion process, a volume of a mixture of 1% formic acid and 10% acetonitrile that is equal to the sample volume was added, and the samples were incubated at 37 °C for 45 min to remove the RapiGest from the solution by precipitation [10,34]. The samples were spun at 16,000× *g* for 10 min and the supernatant was transferred to a fresh insert vial. Prior to LC–MS/MS, samples were spiked by 20 µL of labeled peptides (as internal standards).

Lamin A expression level was calculated by subtracting the protein expression level of lamin A/C from the protein expression levels of various lamin splice variants (lamin C, lamin Δ10, and lamin Δ50) as follows:

lamin A = lamin A/C − (lamin C + lamin Δ10 + lamin Δ50)

### 4.4. Validation of Lamin A/C Gene Expression by RT-qPCR

RT-qPCR assays for *lamin A*, *lamin C*, *lamin AΔ10*, and *lamin AΔ50* splice variants were performed as described previously (Aljada et al., 2016). Total RNA was isolated using the Ambion Aqueous kit (Ambion, Foster City, CA, USA). All isolated RNA samples were treated with DNase I to remove contaminating genomic DNA. The quality and quantity of the isolated RNA were determined using an Agilent Bioanalyzer 2100. One microgram of total RNA was reverse-transcribed using a first strand cDNA synthesis Kit (Millipore, Bedford, MA, USA) followed by RT-qPCR, which was performed using a 7900HT Fast Real-Time PCR System (Applied Biosystems, Foster city, CA, USA), using 2 µL of cDNA, 10 µL of 2× Master mix (150 mM Tris, pH 9.2, 40 mM (NH_4_)_2_SO_4_, 5 mM MgCl_2_, 0.02% Tween-20, 0.4 mM dNTPs, and 1.25 Units Taq Polymerase) and 0.5 µL of 20 μM gene-specific primers. All probes were labeled with a fluorescent dye (FAM) and a fluorescent quencher (BHQ-1), and were purchased from Bio-Basic Canada Inc. (Ontario, QC, Canada). Although normalization to Cyclophilin A was performed, RPL13 and Ubiquitin C showed similar trends and all values were normalized to Cyclophilin A (Table 7). The 2^−ΔΔ*CT*^ method was used for relative quantification of RT-qPCR experiments [14].

### 4.5. Statistical Analysis

Statistical analyses were performed using SigmaStat software version 3.5 (Systat Software, San Jose, CA, USA). Results from quantitative experiments were expressed as mean ± SEM. *p* values < 0.05 were accepted as statistically significant.

## 5. Conclusions

We anticipate that this method will be widely applicable to groups wishing to undertake laminopathic profiling on cells or tissues. We note that ion pairing agents, such as tributylamine, are often avoided for use in positive mode LC/MS due to their potential of ion suppression. In this application on a triple quadrupole instrument, we observed excellent sensitivity and designed our transitions to avoid detection of the tributylamine ion.

## Figures and Tables

**Table 1:** Gradient conditions used for the LC–MS/MS analysis.

Total Time (min)	Flow Rate (mL/min)	% A	% B
**Initial**	0.200	90.0	10.0
1.00	0.200	90.0	10.0
1.01	0.200	90.0	10.0
6.00	0.200	10.0	90.0
6.20	0.200	10.0	90.0
6.50	0.200	90.0	10.0
10.00	0.200	90.0	10.0

**Table 2:** Optimized lamin transitions for all four different peptides, with ion mode (ES+).

Compound	Parent or Precursor (*m*/*z*)	Daughter or Product (*m*/*z*)	Cone Voltage (V)	Collision Energy (V)	Retention Time
lamin A/C ^a^	583.4000	70.1100	44	68	3.771
		91.1000	44	84	3.775
		136.1000	44	44	3.741
lamin A/C ^b^	589.5000	86.2000	46	54	3.741
		90.2000	46	90	3.741
		136.2000	46	42	3.740
lamin C ^a^	484.4000	72.1100	16	50	3.801
		84.0700	16	50	3.801
		110.6000	16	40	3.804
lamin C ^b^	587.4000	80.3000	16	46	3.805
		102.2000	16	40	3.801
		110.2000	16	54	3.801
lamin A Δ10 ^a^	856.4000	86.2000	52	54	3.861
		110.2000	52	62	3.861
		173.2000	52	42	3.861
lamin A Δ10 ^b^	858.5000	70.2000	8	96	3.861
		86.2000	8	54	3.861
		110.2000	8	72	3.861
lamin A Δ50 ^a^	798.5000	60.2000	26	108	4.095
		70.2000	26	82	4.094
		86.2000	26	48	4.094
lamin A Δ50 ^b^	803.5000	60.2000	26	96	4.095
		70.2000	26	78	4.095
		86.2000	26	40	4.094

^a^: Unlabeled peptide, ^b^: Labeled peptide, * Quan product daughter.

**Table 3:** Intra- and inter-day assays showed recorded values (RC), accuracy (RE%), and precision (CV%) of developed lamin method by LC–MS/MS assay.

**Intra-Day Validation (*n* = 6)**													
**Day Validation**	**The Signature Peptide of Transcript Variant**	**150 ng/mL**				**300 ng/mL**				**600 ng/mL**			
		**Mean**	**SD**	**Precision (CV%)**	**Accuracy (%)**	**Mean**	**SD**	**Precision (CV%)**	**Accuracy (%)**	**Mean**	**SD**	**Precision (CV%)**	**Accuracy (%)**
Day1	lamin A/C	153.00	10.61	6.94	102.00	312.00	8.03	7.72	104.00	602.18	10.16	1.68	100.36
	lamin C	144.48	26.55	14.38	96.32	268.46	15.72	5.85	89.48	594.90	7.39	1.24	99.15
	lamin AΔ10	161.35	12.34	7.64	107.56	312.78	8.80	2.81	104.26	631.75	36.65	5.80	105.29
	lamin AΔ50	146.15	10.31	7.05	97.43	307.75	9.28	3.01	102.58	600.61	11.30	1.88	100.10
Day2	lamin A/C	143.92	8.88	6.17	95.94	274.38	13.75	5.01	91.46	526.73	28.82	5.47	87.79
	lamin C	155.87	9.39	6.03	103.91	290.53	4.60	1.58	96.84	620.95	14.92	2.40	103.49
	lamin AΔ10	138.07	16.23	11.75	92.04	293.57	12.30	4.19	97.86	580.78	22.46	3.87	96.80
	lamin AΔ50	156.90	5.56	3.54	104.60	305.72	6.49	2.12	101.91	602.60	4.56	0.76	100.43
Day3	lamin A/C	152.75	21.68	14.19	101.83	295.10	29.19	9.89	98.37	608.78	76.04	12.49	101.46
	lamin C	139.45	5.45	3.91	92.97	272.02	21.50	7.90	90.67	586.33	11.21	1.91	97.72
	lamin AΔ10	151.10	8.61	5.69	100.73	295.50	12.99	4.40	98.50	586.33	10.69	1.82	97.72
	lamin AΔ50	151.65	2.30	1.52	101.10	302.75	13.00	4.29	100.92	602.73	11.29	1.87	100.46
**Inter-Day Validation (*n* = 18)**													
**The Signature Peptide of Transcript Variant**		**150 ng/mL**				**300 ng/mL**				**600 ng/mL**			
		**Mean**	**SD**	**Precision (CV%)**	**Accuracy (%)**	**Mean**	**SD**	**Precision (CV%)**	**Accuracy (%)**	**Mean**	**SD**	**Precision (CV%)**	**Accuracy (%)**
lamin A/C		149.89	14.61	9.75	99.93	290.49	21.70	7.47	96.83	579.23	58.67	10.13	96.54
lamin C		146.60	17.09	11.66	97.73	277.01	17.72	6.40	92.34	599.96	18.94	3.16	99.99
lamin AΔ10		159.60	15.50	9.71	106.40	300.62	14.00	4.66	100.21	599.62	33.60	5.60	99.94
lamin AΔ50		148.98	6.88	4.62	99.32	305.41	9.59	3.14	101.80	601.98	9.06	1.51	100.33
lamin A/C		149.89	14.61	9.75	99.93	290.49	21.70	7.47	96.83	579.23	58.67	10.13	96.54

**Table 4:** Linearity and sensitivity (*n* = 3).

The Signature Peptide of Transcript Variant	Linearity (*n* = 3)				Sensitivity (*n* = 3)				
	Slope	SD	Intercept	R	LLOQ (nM)	Mean	SD	Precision (CV%)	Accuracy (%)
lamin A/C	0.0026	0.0005	0.0089	0.9969	50.00	52.37	7.06	13.48	104.73
lamin C	0.0003	0.0002	−0.0223	0.9726	50.00	48.47	6.05	12.48	96.93
lamin AΔ10	0.0032	0.0008	−0.0428	0.9917	50.00	60.20	8.80	14.62	120.40
lamin AΔ50	0.0032	0.0018	−0.0091	0.9933	50.00	44.23	5.26	11.89	88.47

**Table 5:** Stability of peptides in extracted samples (*n* = 9).

Stability (%)					
Storage Temp (°C)	ng/mL	The Signature Peptide of Transcript Variant			
		lamin A/C	lamin C	lamin AΔ10	lamin AΔ50
Fresh (−80 °C)	150	100	100	100	100
	300	100	100	100	100
	600	100	100	100	100
2 weeks (−20 °C)	150	100.65	101.89	99.17	100.21
	300	99.97	97.45	99.76	99.59
	600	99.28	100.09	99.64	99.94
4 weeks (−20 °C)	150	98.06	98.27	98.76	96.99
	300	97.46	96.45	98.26	96.44
	600	99.22	99.86	100.07	99.31
1 week (4 °C)	150	97.59	98.95	97.56	96.97
	300	98.25	96.3	96.14	95.87
	600	97.85	99.07	100.03	99.802
1 week (RT)	150	87.75	86.56	79.52	80.37
	300	86.99	92.55	93.58	83.69
	600	95.6	96.77	98.99	95.65

**Table 6:** Amino acids sequences of *lamin A/C* gene transcript variant signature peptides.

Transcript Variant	Sequence Position	NCBI Reference	Signature Peptide Sequence
**lamin A/C (Total)**	AA78–89; exon 1	NM_170707.3	AAYEAELGDAR AAYEAELGDAR (A = d3-Ala) *
**lamin C**	AA547–572; in the junction region between the end of exon 10 with its specific 5 amino acids	NM_005572.3	SVTVVEDDEDEDGDDLLHHHHVSGSR SVTVVEDDEDEDGDDLLHHHHVSGSR (V = d8-Val) *
**lamin AΔ10**	AA529–553; in the junction between exon 9 and exon 11	NM_170708.3	TALINSTGEGSHCSSSGDPAEYNLR TALINSTGEGSHCSSSGDPAEYNLR (A = d3-Ala) *
**lamin AΔ50**	AA599–615; in the junction region of the exonic cryptic site of exon 11 which lacks the terminal 150 amino acids and exon 12	NM_001282626.1	ASASGSGAQSPQNCSIM ASASGSGAQSPQNCSIM (A = d3-Ala) *

* Internal standard.

**Table 7:** Primer/probe sequences used for RT-qPCR.

	Probe	Primer
Lamin A	5′-CGCTGAGTACAACCT -3′	F 5′- GACGAGGATGAGGATGGAGA-3′ R 5′-GAGTGACCGTGACACTGGAG-3′
Lamin C	5′-AGATGACCTGCTCCATCACC-3′	F 5′-GTGGAAGGCACAGAACACCT-3′ R 5′-GCGGCGGCTACCACTCAC-3′
Lamin AΔ10	5′-AGTACAACCTGCGCTCGCGC-3′	F 5′-AACTCCACTGGGGAAGGCTCC-3′ R 5′-GCTCCTGAGCCGCTGGCAGA-3′
Lamin AΔ50	5′-AGCATCATGTAATCTGGGACCT-3′	F 5′-GCGTCAGGAGCCCTGAGC-3′ R 5′-GACGCAGGAAGCCTCCAC-3′
Ubiquitin	5′-CCCACCTCTGAGACGGAGCACCAG-3′	F 5′-ACTACAACATCCAGAAAGAGTCCA-3′ R 5′- CCAGTCAGGGTCTTCACGAAG-3′
RPL-13	5′-CGCAAGCGGATGAACACCAACCCT-3′	F 5′-AACAAGTTGAAGTACCTGGCTTTC-3′ R 5′-TGGTTTTGTGGGGCAGCATA-3′
Cyclophilin A	5′-ACAGCTCAAAGGAGACGCGGCCCA-3′	F 5′-CCCACCGTGTTCTTCGACAT-3′ R 5′-TTTCTGCTGTCTTTGGGACCTT-3′

## Abbreviations

°C	Degrees Celsius
µg/mL	microgram/milliliter
C-terminus	Carboxyl terminus
CV	Coefficient of Variation
DNA	Deoxyribonucleic acid
FBS	Fetal bovine serum
GFP	Green Fluorescent Protein
HQC	High Quality control
IS	Internal Standard
L/mL/µL	Liter/milliliter/microliter
LC–MS/MS	Liquid Chromatography Mass Spectrometry MS
TV2	lamin C
TV3	lamin AΔ10
TV4	lamin AΔ50
LQC	Low Quality control
M/mM/µM	Molar/millimolar/micromolar
mRNA	messenger RNA
ng/mL	nanogram/milliliter
PBS	Phosphate buffered saline
PCR	Polymerase chain reaction
QC	Quality control
qPCR	Quantitative PCR
RE	Relative Error
REC%	recovery percent
RNA	Ribonucleic acid
